# Langerhans Cells Sense *Staphylococcus aureus* Wall Teichoic Acid through Langerin To Induce Inflammatory Responses

**DOI:** 10.1128/mBio.00330-19

**Published:** 2019-05-14

**Authors:** Rob van Dalen, Jacinto S. De La Cruz Diaz, Matevž Rumpret, Felix F. Fuchsberger, Nienke H. van Teijlingen, Jonas Hanske, Christoph Rademacher, Teunis B. H. Geijtenbeek, Jos A. G. van Strijp, Christopher Weidenmaier, Andreas Peschel, Daniel H. Kaplan, Nina M. van Sorge

**Affiliations:** a Medical Microbiology, University Medical Center Utrecht, Utrecht University, Utrecht, The Netherlands; b Departments of Dermatology and Immunology, University of Pittsburgh, Pittsburgh, Pennsylvania, USA; c Department of Biomolecular Systems, Max Planck Institute of Colloids and Interfaces, Potsdam, Germany; d Department of Experimental Immunology, Academic Medical Center, University of Amsterdam, Amsterdam, The Netherlands; e Interfaculty Institute of Microbiology and Infection Medicine, University of Tübingen, Tübingen, Germany; f German Center for Infection Research (DZIF), Tübingen, Germany; Imperial College London; Institut Pasteur

**Keywords:** atopic dermatitis, glycosylation, Langerhans cell, langerin, Staphylococcus aureus, wall teichoic acid

## Abstract

Staphylococcus aureus is a major cause of skin and soft tissue infections and aggravator of the inflammatory skin disease atopic dermatitis (AD [eczema]). Epicutaneous exposure to S. aureus induces Th17 responses through skin Langerhans cells (LCs), which paradoxically contribute to host defense but also to AD pathogenesis. The molecular mechanisms underlying the interaction between S. aureus and LCs are poorly understood. Here we demonstrate that human LCs directly interact with S. aureus through the pattern recognition receptor langerin (CD207). Human, but not mouse, langerin interacts with S. aureus through the conserved β-*N-*acetylglucosamine (GlcNAc) modifications on wall teichoic acid (WTA), thereby discriminating S. aureus from other staphylococcal species. Importantly, the specific S. aureus WTA glycoprofile strongly influences the level of proinflammatory cytokines that are produced by *in vitro*-generated LCs. Finally, in a murine epicutaneous infection model, S. aureus strongly upregulated transcripts of *Cxcl1*, *Il6*, and *Il17*, which required the presence of both human langerin and WTA β-GlcNAc. Our findings provide molecular insight into the unique proinflammatory capacities of S. aureus in relation to skin inflammation.

## Abstract (precis)

The bacterium Staphylococcus aureus is an important cause of skin infections and is also associated with the occurrence and severity of eczema. Langerhans cells (LCs), a specific subset of skin immune cells, participate in the immune response to S. aureus, but it is yet unclear how LCs recognize S. aureus. Therefore, we investigated the molecular mechanism underlying the interaction between LCs and S. aureus. We identified that wall teichoic acid, an abundant polymer on the S. aureus surface, is recognized by langerin, a receptor unique to LCs. This interaction allows LCs to discriminate S. aureus from other related staphylococcal species and initiates a proinflammatory response similar to that observed in patients with eczema. Our data therefore provide important new insights into the relationship between S. aureus, LCs, and eczema.

## INTRODUCTION

Staphylococcus aureus is an important cause of skin and soft tissue infections and is strongly associated with the inflammatory skin disease atopic dermatitis (AD [also known as eczema]), which affects up to 20% of children and 3% of adults worldwide (1–5). Langerhans cells (LCs) are key sentinel cells in the skin epidermis and are implicated in S. aureus-induced skin inflammation. LCs are equipped with a diverse set of pattern recognition receptors (PRRs) to sense intruders, including the LC-specific C-type lectin receptor (CLR) langerin (CD207) (6). LCs can phagocytose microbes and initiate adaptive immune responses by activating skin-resident immune memory cells or naive immune cells in the lymph nodes (7, 8). In response to S. aureus, murine LCs induce Th17 responses that help to contain S. aureus infection but paradoxically also aggravate AD (9, 10). Despite the functional importance of LCs in S. aureus-mediated skin pathology, the molecular interaction between LCs and S. aureus and the functional response of LCs have received little attention.

A dominant and evolutionarily conserved component of the S. aureus surface is wall teichoic acid (WTA), which is important in nasal colonization, S. aureus-induced endocarditis, β-lactam resistance, and phage-mediated horizontal gene transfer (11–15). In the majority of S. aureus lineages, WTA is composed of 20 to 40 ribitol phosphate (RboP) repeating units modified with d-alanine and *N-*acetylglucosamine (GlcNAc). GlcNAc is *O*-linked to the C-4 hydroxyl of RboP in either the α or β configuration by glycosyltransferases TarM and TarS, respectively (13, 16). Several S. aureus WTA glycoprofiles can be discriminated: WTA β-GlcNAcylation is conserved in almost all S. aureus strains, whereas WTA α-GlcNAcylation is only present in about one-third of the S. aureus isolates. A small selection of isolates even completely lack WTA glycosylation (11, 17). Finally, WTA of S. aureus lineage ST395 is composed of a glycerol phosphate (GroP) backbone modified by *N*-acetylgalactosamine (GalNAc) (15). WTA glycosylation is an important determinant in host-pathogen interactions, which include attachment to scavenger receptor SREC-1 in the nasal epithelium and opsonization by antibodies and mannose-binding lectin (18–20).

We demonstrate an important role of the PRR langerin in sensing the β-GlcNAc epitope on S. aureus WTA, which explains the lack of binding to other non-AD-associated staphylococcal species. Interestingly, simultaneous decoration of WTA with α-GlcNAc impairs langerin interaction and dampens cytokine responses of LCs, implying that S. aureus can modulate immune detection and subsequent inflammation in the epidermis. Murine infection experiments confirmed that the interaction of langerin with WTA β-GlcNAc contributes to enhanced skin inflammation, most prominently interleukin-17 (IL-17) production. In conclusion, we identify WTA β-GlcNAc as an important molecular trigger for S. aureus-induced skin inflammation through the interaction with LC-expressed langerin.

## RESULTS

### Langerin is a receptor for S. aureus on human LCs.

**FIG 1:**
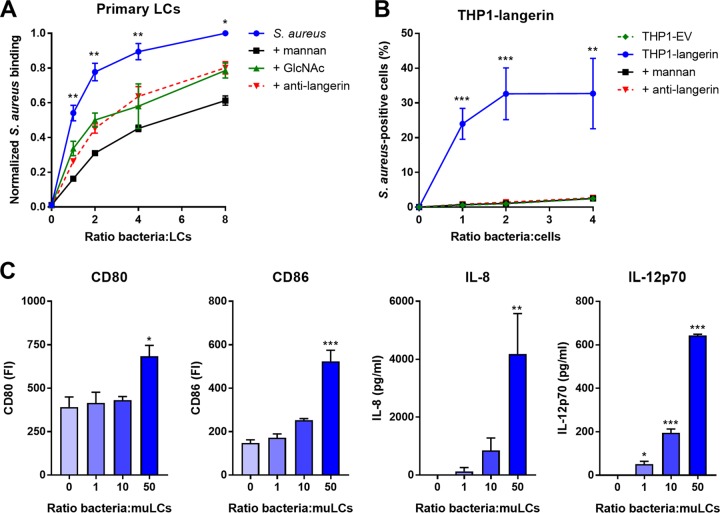
Langerin is a receptor for S. aureus on human LCs. (A) Binding of GFP-expressing S. aureus Newman to isolated primary human LCs from four different donors. The interaction was blocked by addition of mannan (*n* = 4), GlcNAc (*n* = 4), or anti-langerin blocking antibody (*n* = 2; using GFP-expressing S. aureus Newman Δ*spa* Δ*sbi*). Data were pooled and normalized to the maximum binding level observed in each donor (ratio 8) and are presented as mean ± standard error of mean (SEM). Indicated statistical differences refer to the blocking conditions compared to the nonblocked control. (B) Binding of S. aureus to THP1-langerin cells. Human langerin-transduced or empty vector (EV)-transduced THP1 cells were incubated with different amounts of GFP-expressing S. aureus Newman Δ*spa* Δ*sbi*. The interaction was blocked by addition of mannan or anti-langerin blocking antibody. Data are presented as percentage GFP-positive cells ± SEM from three independent experiments. (C) Expression of costimulatory molecules CD80 and CD86 and production of cytokines IL-8 and IL-12p70 by muLCs after stimulation with gamma-irradiated S. aureus USA300. Mean concentrations ± SEM from three independent experiments are shown. *, *P* < 0.05; **, *P* < 0.01; ***, *P* < 0.001.

The molecular interaction between LCs and S. aureus has received little attention. We therefore investigated whether LCs and S. aureus interact directly by incubating primary LCs isolated from human skin with green fluorescent protein (GFP)-expressing S. aureus. LCs from four different donors bound S. aureus in a dose-dependent manner (Fig. 1A). The levels at which the interaction was saturated varied between the donors from approximately 40% (donor 1) to 80% (donor 3) of S. aureus-positive LCs (see Fig. S1 in the supplemental material). To investigate the identity of interacting receptors on LCs, we preincubated LCs with mannan, a ligand for many PRRs of the CLR family. Depending on the bacterium-to-cell ratio, S. aureus binding was reduced by 35 to 70% compared to nonblocking conditions in all donors (Fig. 1A). Similarly, the interaction was inhibited by approximately 35% by preincubation of LCs with the monosaccharide GlcNAc (Fig. 1A). Langerin is a mannan- and GlcNAc-specific CLR that is exclusively expressed on LCs. We therefore investigated whether langerin would be involved in interaction with S. aureus. Indeed, preincubation with an anti-langerin blocking antibody reduced binding of *spa-* and *sbi*-deficient (to prevent Fc-dependent antibody binding) S. aureus in donors 3 and 4 by on average 35% compared to the control, depending on the infective dose (Fig. 1A; Fig. S1). To confirm involvement of langerin in the interaction between S. aureus and LCs, we introduced langerin in the THP1 cell line, which normally does not express langerin. Transduction of langerin, but not of empty vector (EV), conferred S. aureus binding to THP1 cells, which could be completely inhibited by addition of mannan or anti-langerin blocking antibody (Fig. 1B).

10.1128/mBio.00330-19.1 FIG S1 S. aureus binding to human LCs. Shown is binding of S. aureus to isolated primary human LCs from four different donors. LCs from donors 1 and 2 were incubated with GFP-expressing S. aureus Newman, and LCs from donors 3 and 4 were incubated with GFP-expressing S. aureus Newman Δ*spa* Δ*sbi* (to avoid Fc-dependent binding to the anti-langerin blocking antibody). The interaction was blocked by addition of mannan, GlcNAc, or anti-langerin blocking antibody (donors 3 and 4 only). Download FIG S1, TIF file, 0.5 MB. Copyright © 2019 van Dalen et al. 2019 van Dalen et al. This content is distributed under the terms of the Creative Commons Attribution 4.0 International license.

S. aureus-exposed LCs were previously demonstrated to initiate T cell proliferation (21). However, the functional responses of LCs have not been assessed in these experiments. Therefore, we stimulated MUTZ-3-derived LCs (muLCs), a well-established cell model for human LCs (22, 23), with S. aureus and measured muLC activation through expression of costimulatory molecules and cytokine production after 24 h. For these experiments, S. aureus was gamma irradiated to prevent toxin-mediated cell lysis (24). muLCs upregulated expression of costimulatory molecules CD80 and CD86 and produced significant amounts of IL-8 and IL-12p70 in a dose-dependent response to S. aureus (Fig. 1C). Together, these data demonstrate that LCs respond to S. aureus and that langerin is an important innate PRR for S. aureus on human LCs.

### Langerin specifically recognizes S. aureus in a *tarS*-dependent manner through the conserved WTA β-GlcNAc epitope.

**FIG 2:**
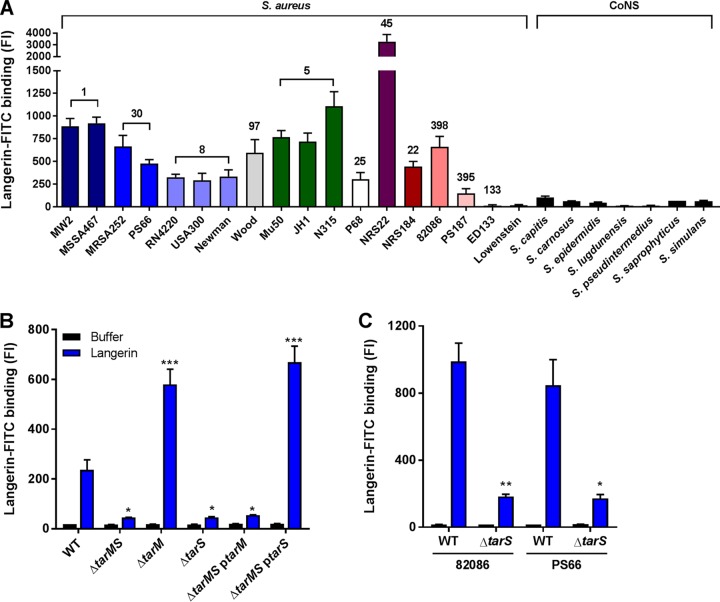
Langerin specifically recognizes S. aureus in a *tarS*-dependent manner through the conserved WTA β-GlcNAc epitope. Shown is binding of recombinant human langerin-FITC to (A) 18 wild-type S. aureus strains (11 different clonal complexes, indicated above the bars and by different colors) and a selection of coagulase-negative staphylococcal species (CoNS), (B) the S. aureus USA300 wild-type (WT) and WTA biosynthesis Δ*tarMS*, Δ*tarM*, Δ*tarS*, Δ*tarMS* p*tarM*, and Δ*tarMS* p*tarS* strains, and (C) two representative S. aureus isolates (82086 and PS66) that naturally lack *tarM* and their isogenic Δ*tarS* mutants. Data are presented as geometric mean fluorescence intensity ± SEM from three independent experiments. *, *P* < 0.05; **, *P* < 0.01; ***, *P* < 0.001.

To further investigate langerin’s interaction with staphylococci, we tested binding of a fluorescein isothiocyanate (FITC)-labeled trimeric construct of the extracellular domains of human langerin (langerin-FITC) to a broader collection of 18 S. aureus strains from 11 different clonal complexes, as well as several coagulase-negative staphylococci (CoNS). Langerin-FITC bound to most tested S. aureus strains but to none of the CoNS species (Fig. 2A), indicating that langerin interacts with a ligand that is specific for and highly conserved in S. aureus.

Since langerin is a CLR with affinity for carbohydrates, it is likely that it interacts with glycosylated structures on the S. aureus surface. The S. aureus glycome includes glycosylated proteins, capsular polysaccharide, poly-β(1-6)-*N*-acetylglucosamine (PNAG), and WTA (25). Interestingly, the three tested S. aureus strains that showed no or only low-level binding of langerin-FITC (ED133, Lowenstein, and PS187 [Fig. 2A]) differ from the other tested S. aureus strains in the structural composition of WTA. ED133 and Lowenstein completely lack WTA GlcNAcylation, whereas PS187 belongs to the ST395 lineage that expresses GroP-GalNAc WTA (15, 17, 26). Given the high density of WTA on the S. aureus surface and apparent correlation between langerin interaction and WTA structure, we hypothesized that WTA GlcNAc modifications are likely candidates for the interaction with langerin.

To test this hypothesis, we assessed binding of langerin-FITC to a panel of S. aureus knockout strains that lack glycosyltransferases TarM and TarS, which modify WTA with α-GlcNAc and β-GlcNAc, respectively. Loss of both glycosyltransferases (Δ*tarMS*) reduced langerin-FITC binding to S. aureus by 70 to 85%, depending on the S. aureus strain background (Fig. 2B; see Fig. S2A and B in the supplemental material), demonstrating that WTA GlcNAc is a major target of langerin. To investigate whether langerin specifically recognized either α-GlcNAc or β-GlcNAc, we tested the individual *tarM* and *tarS* knockout strains as well as the Δ*tarMS* mutant complemented with either *tarM* or *tarS* on an expression plasmid (Δ*tarMS* p*tarM* and Δ*tarMS* p*tarS*). Langerin-FITC only bound to S. aureus strains that expressed β-GlcNAc, whereas α-GlcNAc was dispensable for binding (Fig. 2B; Fig. S2A and B). Similarly, langerin-FITC binding to S. aureus strains 82086 and PS66, which are naturally deficient for WTA α-GlcNAc, was reduced by 80% in isogenic Δ*tarS* strains (Fig. 2C). These results show that langerin interacts with S. aureus in a *tarS*-dependent manner and provide the first demonstration of an anomeric specific interaction of a human innate receptor with a Gram-positive surface polysaccharide.

10.1128/mBio.00330-19.2 FIG S2 Langerin-FITC interaction with S. aureus *tarM*/*tarS* mutants in different genetic backgrounds. Shown is binding of recombinant human langerin-FITC to (A) the S. aureus RN4220 WT or Δ*tarMS*, Δ*tarM*, Δ*tarS*, Δ*tarMS* p*tarM*, and Δ*tarMS* p*tarS* mutant strains and (B) the Newman WT or Δ*tarMS*, Δ*tarMS* p*tarM*, and Δ*tarMS* p*tarS* mutant strains. Data are presented as geometric mean fluorescence intensity ± SEM from three independent experiments. **, *P* < 0.01; ***, *P* < 0.001. Download FIG S2, TIF file, 0.2 MB. Copyright © 2019 van Dalen et al. 2019 van Dalen et al. This content is distributed under the terms of the Creative Commons Attribution 4.0 International license.

Although α-GlcNAc is not the target of langerin, its presence or absence influenced the level of langerin-FITC binding: mutant strains lacking *tarM* (Δ*tarM* and Δ*tarMS* p*tarS*) showed significantly increased binding compared to the wild type (WT) (Fig. 2B; Fig. S2A and B). Chemical analysis of the WTA composition of strain RN4220 Δ*tarM* by Kurokawa et al. suggests a similar amount of β-GlcNAcylation compared to the wild-type strain (19). Therefore, the enhanced langerin-FITC binding we observed is likely not caused by increased WTA β-GlcNAcylation but potentially results from reduced steric hindrance by α-GlcNAc.

As S. aureus expresses many human-specific adhesins and immune evasion factors (27), we investigated the interaction with murine langerin-FITC, which shares 76% identity with the human langerin-FITC construct (28). Binding of murine langerin-FITC to S. aureus was detectable, but was 10- to 100-fold lower than that of human langerin (see Fig. S3 in the supplemental material). The 50% effective concentration (EC_50_) of human langerin-FITC for S. aureus USA300 was 9.7 μg/ml (range, 8.3 to 11.3 μg/ml), while binding of murine langerin-FITC was not yet saturated at 50 μg/ml. Despite low level and nonsaturable binding, murine langerin interaction with S. aureus could be blocked by addition of mannan, suggesting that the interaction is specific. Altogether, this indicates that the langerin-S. aureus interaction has a certain degree of species specificity.

10.1128/mBio.00330-19.3 FIG S3 Differential interaction of human and mouse langerin-FITC with S. aureus. Shown is binding of recombinant human langerin-FITC (blue) and mouse langerin-FITC (red) to wild-type S. aureus strains USA300, RN4220, and Newman. At all concentrations, human langerin-FITC binding was compared to mouse langerin-FITC binding to the same strain. Data are presented as geometric mean fluorescence intensity ± SEM from three independent experiments. **, *P* < 0.01; ***, *P* < 0.001. Download FIG S3, TIF file, 0.09 MB. Copyright © 2019 van Dalen et al. 2019 van Dalen et al. This content is distributed under the terms of the Creative Commons Attribution 4.0 International license.

### The S. aureus WTA glycoprofile affects the proinflammatory cytokine response of LCs.

**FIG 3:**
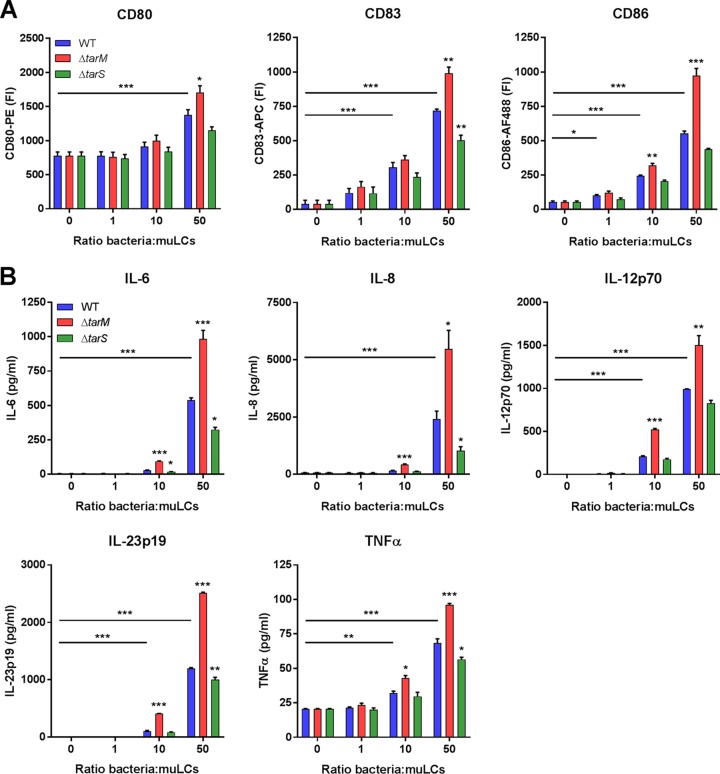
The S. aureus WTA glycoprofile affects the proinflammatory cytokine response of LCs. (A) Expression of costimulatory molecules CD80 and CD86 and maturation marker CD83 and (B) production of cytokines IL-6, IL-8, IL12p70, IL23p19, and TNF-α by muLCs upon incubation with gamma-irradiated S. aureus USA300 wild-type (WT) or Δ*tarM* or Δ*tarS* mutant cells. muLCs stimulated with WT S. aureus were compared to unstimulated controls, and muLCs stimulated with the Δ*tarM* and Δ*tarS* mutants were compared to their respective WT controls within the same ratio. IL-4, IL-10, and IFN-γ concentrations were assessed, but were below the detection limit. Data are presented as geometric mean fluorescence intensity or mean concentration ± SEM from three independent experiments. *, *P* < 0.05; **, *P* < 0.01; ***, *P* < 0.001.

Given the importance of langerin for interaction between S. aureus and LCs, we investigated whether distinct WTA GlcNAc glycoprofiles influenced the muLC response at the level of costimulatory molecules and cytokine expression. In line with our initial observations, stimulation of muLCs with wild-type gamma-irradiated S. aureus upregulated expression of activation markers CD80, CD83, and CD86 (Fig. 1D and 3A). Stimulation with the β-GlcNAc-deficient S. aureus Δ*tarS* mutant reduced expression of these markers compared to the wild type, whereas stimulation with the α-GlcNAc-deficient S. aureus Δ*tarM* mutant significantly enhanced expression at higher multiplicities of infection (MOI) (Fig. 3A). In addition, muLCs secreted significant levels of IL-6, IL-8 IL-12p70, IL-23p19, and tumor necrosis factor alpha (TNF-α) (Fig. 3B), but not IL-4, gamma interferon (IFN-γ), or anti-inflammatory IL-10 (all below the detection limit), in response to S. aureus. Overall, cytokine levels were reduced after muLC stimulation with the S. aureus Δ*tarS* mutant compared to the WT, whereas stimulation with the S. aureus Δ*tarM* mutant significantly increased the secretion of these cytokines (Fig. 3B). These functional differences correspond to the observed differences in binding of recombinant langerin-FITC to the S. aureus WT and Δ*tarM* and Δ*tarS* mutant strains (Fig. 2B). Overall, these data indicate that the proinflammatory cytokine response of LCs is strongly influenced by the S. aureus WTA glycoprofile.

### Epicutaneous infection with S. aureus induces skin inflammation that requires both human langerin and WTA β-GlcNAc expression.

**FIG 4:**
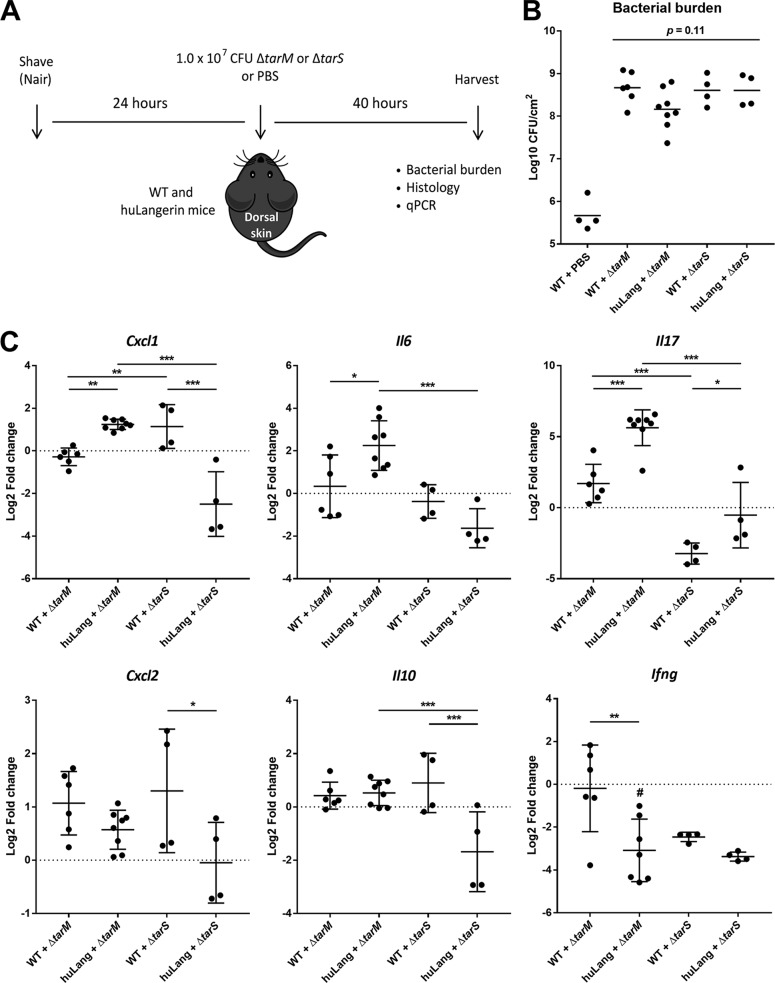
Epicutaneous infection with S. aureus induces skin inflammation that requires both human langerin and WTA β-GlcNAc expression. (A) Experimental design for epicutaneous infection of WT mice with the S. aureus Δ*tarM* mutant (*n* = 6), huLangerin mice with the S. aureus Δ*tarM* mutant (*n* = 8), WT mice with the S. aureus Δ*tarS* mutant (*n* = 4), huLangerin mice with the S. aureus Δ*tarS* mutant (*n* = 4), and WT mice with PBS controls (*n* = 4). (B) Bacterial burden of the lesions 40 h postinoculation, presented as log_10_-transformed CFU/cm^2^ ± SD. (C) Transcript abundance of *Cxcl1*, *Cxcl2*, *Il6*, *Il17*, *Il10*, and *Ifng* in whole-skin homogenates 40 h post-epicutaneous inoculation. Data are presented as log_2_-transformed fold change ± standard deviation (SD) relative to *Gapdh* and normalized to the mean value of the group WT mice plus PBS. *, *P* < 0.05; **, *P* < 0.01; ***, *P* < 0.001. #, no *C_T_* value was reached for *Ifng* in one sample.

Given the observed species specificity of langerin for S. aureus WTA β-GlcNAc (Fig. S2A), we used human langerin-diphtheria toxin receptor (huLangerin-DTR) mice, which constitutively express human langerin on mouse LCs, as a huLangerin transgenic mouse model (29). Wild-type (WT) and huLangerin mice were epicutaneously inoculated with 10^7^ CFU of the S. aureus Δ*tarM* or Δ*tarS* mutant (9, 30) (Fig. 4A). Since we do not know how WTA glycosylation is regulated in the context of the skin, we used the genetically stable Δ*tarS* and Δ*tarM* mutant strains, thereby maximizing the interaction of human langerin with the Δ*tarM* mutant, while it can no longer be engaged by the Δ*tarS* mutant. At the time of sacrifice and skin collection, no consistent differences between the infected groups were observed either macroscopically or microscopically (see Fig. S4A and B in the supplemental material). Also bacterial burdens in the skin did not significantly differ between the groups, although there was a trend toward lower CFU in the huLangerin Δ*tarM* mutant-infected group, compared to the other S. aureus-infected groups (Fig. 4B; *P* = 0.11). In contrast, we observed significantly enhanced expression of *Cxcl1* (KC), *Il6*, and *Il17*, but not of *Cxcl2* (MIP-2), *Ifng*, or *Il10*, in the huLangerin group as opposed to WT controls after infection with the S. aureus Δ*tarM* mutant (Fig. 4C). Importantly, this inflammatory response was not observed in huLangerin mice infected with the S. aureus Δ*tarS* mutant, suggesting that this response was specific for the interaction between huLangerin and WTA β-GlcNAc (Fig. 4C). In contrast, *Ifng* was downregulated in the huLangerin group infected with the Δ*tarM* mutant as well as in both Δ*tarS* mutant-infected groups, indicating an absence of Th1 stimulation. These results corroborate the observed *in vitro* responses of muLCs to S. aureus stimulation (Fig. 3) and provide the first *in vivo* demonstration of the involvement of human langerin in the skin immune response to S. aureus, which strongly depends on recognition of WTA β-GlcNAc.

10.1128/mBio.00330-19.4 FIG S4 Macroscopy and microscopy of mouse skin upon epicutaneous infection with S. aureus. (A) Photos of infection sites of all individual mice in the five groups 40 h post-epicutaneous inoculation, with sexes indicated by M (male) or F (female). (B) Representative images of hematoxylin and eosin staining of skin biopsy specimens from each group 40 h post-epicutaneous inoculation. The scale bars represent 50 μm. Download FIG S4, TIF file, 2.5 MB. Copyright © 2019 van Dalen et al. 2019 van Dalen et al. This content is distributed under the terms of the Creative Commons Attribution 4.0 International license.

## DISCUSSION

Despite the emerging role of LCs in S. aureus-mediated skin inflammation, there is limited information on the molecular pathways and functional consequences of LC-S. aureus interaction. We observed that S. aureus triggers LCs to produce inflammatory cytokines, which are known be important for the induction of Th17-polarized immune responses. This complements findings by others, who have demonstrated that LCs internalize S. aureus and subsequently induce inflammatory Th17 responses (9, 10, 21, 31). We did not observe production of Th1- or Th2-polarizing cytokines, although the involvement of both Th1 and Th2 cells has been well described in chronic AD (32). It is likely that the initiation of Th2 responses in AD is mediated by cell types other than LCs, such as follicular T helper cells, which were shown to be responsible for the initial production of IL-4 and drive Th2 expansion (33).

Detection of S. aureus WTA β-GlcNAc is of critical importance for the induced LC cytokine response and is affected by codecoration with α-GlcNAc, a characteristic of approximately one-third of the S. aureus isolates (11). Similarly, S. aureus was suggested to protect itself from infection by lytic podophages through the ability to regulate its WTA glycoprofile (17). Based on data by others, *tarM* is regulated as part of the GraRS regulon, which is known to control S. aureus susceptibility to antimicrobial host defenses (34, 35). However, whether and how GraRS and WTA GlcNAcylation are affected during skin colonization and infection remain to be determined.

In addition to regulation of glycosylation, WTA abundance can be regulated through *tarH*, the ATPase required for WTA transport across the membrane (36). High WTA expression increased the ability to induce skin abscesses in mice (36). However, these data cannot be directly compared to our study, since mice were infected subcutaneously, thereby bypassing the LCs. In addition, the species specificity of langerin should be taken into account. We demonstrate that mouse langerin shows significantly reduced binding to S. aureus compared to human langerin, underlining previous studies that reported differences in ligand specificity of these orthologs (28).

LCs and langerin were previously implicated in host defense against various other pathogens. LCs internalize and degrade HIV-1 viral particles in a langerin-dependent manner to prevent infection of deeper layers of the mucosa (37, 38). Langerin has also been identified as a major receptor for fungal pathogens on LCs through recognition of mannose and β-glucan structures (39). The Gram-negative bacterium Yersinia pestis is the only other bacterium known to interact with langerin and does so through its lipo-oligosaccharide (40). We identify S. aureus WTA β-GlcNAc as a new ligand for langerin. WTA is an abundant evolutionarily conserved feature on the surface of Gram-positive bacteria, making it advantageous for the host to recognize such structures in a timely manner. Although several receptors for S. aureus WTA have been described, langerin is the first human innate receptor to discriminate between the α-GlcNAc and β-GlcNAc modifications.

As an opportunistic microbial resident of the skin, S. aureus is a frequent cause of skin infections and contributes to the development of inflammatory skin disorders. Therefore, the recognition of S. aureus WTA by strategically localized epidermal LCs may be key to maintaining skin homeostasis and preventing the development of infection or chronic inflammation. Indeed, epicutaneous S. aureus infection of huLangerin mice induced high transcript levels of *Cxcl1*, *Il6*, and *Il17*, which was dependent on the presence of β-GlcNAc on S. aureus WTA. This response shows signatures of Th17 activation, which was previously reported to be important for a protective immune response against S. aureus (9, 10, 21, 31). However, the short time frame of this experiment suggests that the observed *Il17* transcripts may derive from innate γδ T cells, the main producers of IL-17 in mouse skin (41), rather than adaptive Th17 cells, the main producer of IL-17 in human skin. Additional studies are needed to pinpoint the cell type responsible for the observed IL-17 responses. Despite the increased inflammatory response, there was no evident effect on bacterial clearance in our *in vivo* infection experiment. We speculate that the time of sacrifice (i.e., 40 h postinfection) is too early to observe such differences, and future experiments will therefore include analysis of bacterial burden at different times postinfection.

The identification of S. aureus as a new langerin-interacting pathogen is especially interesting in the context of AD. First, S. aureus has been identified as a driver of disease progression in a murine AD model, which depended on the presence of LCs (10). How this relates to the involvement of LCs in human AD remains to be investigated. Second, genome-wide association studies (GWAS) identified *CD207*, the gene encoding langerin, as an AD susceptibility locus (42, 43). In these studies, polymorphisms in a putative enhancer region of *CD207*, which are predicted to increase langerin expression, were protective for AD. Our data now functionally link langerin to S. aureus, which could explain the strong association between S. aureus and AD, as well as the described driver function of S. aureus in AD disease progression. Also our observation that WTA α-GlcNAc attenuates LC activation may be important in the context of AD. The CC1 lineage is particularly overrepresented in isolates from AD skin and was suggested to have unidentified features that enable colonization by and proliferation of S. aureus on AD skin (44). Interestingly, all CC1 strains are *tarM* positive (45), providing the potential to regulate the WTA glycoprofile by codecoration with α-GlcNAc. This could enable the bacteria to skew the inflammatory status of the skin and gain an advantage to colonize AD skin. Our data may provide molecular insight into the association between AD and S. aureus from two different angles: on the immunological side, we show how langerin and LCs are involved in the immune response to S. aureus, while on the microbiological side, the involvement of langerin could explain the association of S. aureus but not CoNS species with AD and possibly also the overrepresentation of *tarM*-bearing CC1 strains in AD.

In conclusion, we identify S. aureus WTA β-GlcNAc as an important molecular trigger for S. aureus-induced skin inflammatory responses through interaction with langerin. Our findings provide a deeper understanding of the specific association of S. aureus with skin inflammation and can help in the development of new treatment strategies for S. aureus-associated skin and soft tissue infections and inflammatory skin diseases.

## MATERIALS AND METHODS

### Ethics statement.

Human skin tissue was collected from healthy anonymous donors undergoing corrective breast or abdominal surgery. In concordance with Dutch law, no informed consent was required, as the tissue collected for this study was exclusively waste material from a standard surgical procedure that had not been altered for the purpose of this study. This study, including the tissue-harvesting procedures, was approved by the Medical Ethics Review Committee of the Academic Medical Center Amsterdam, The Netherlands.

The mouse protocols were approved beforehand under license no. 15096624 by the AAALAC-accredited Institutional Animal Care and Use Committee of the University of Pittsburgh, Pennsylvania, USA, and are adherent to the regulations and guidelines of the United States Animal Welfare Act and Public Health Service Policy.

### Bacterial strains and culture conditions.

S. aureus, *S. capitis*, *S. carnosus*, S. epidermidis, *S. lugdunensis*, S. pseudintermedius, *S. saprophyticus*, and *S. simulans* strains (46–55) (see Table S1 in the supplemental material) were grown overnight at 37°C with agitation in 5 ml Todd-Hewitt broth (THB; Oxoid). For S. aureus strains that were plasmid complemented, THB was supplemented with 10 μg/ml chloramphenicol (Sigma-Aldrich). Overnight cultures were subcultured the next day in fresh THB and grown to an optical density at 600 nm (OD_600_) of 0.4 for *S. capitis* and an OD_600_ of 0.6 to 0.7 for all other bacteria, which correspond to mid-exponential growth phases.

10.1128/mBio.00330-19.5 TABLE S1 Bacterial strains used in this study. Download Table S1, DOCX file, 0.02 MB. Copyright © 2019 van Dalen et al. 2019 van Dalen et al. This content is distributed under the terms of the Creative Commons Attribution 4.0 International license.

### Cell culture and muLC differentiation.

MUTZ-3 cells (ACC-295; DSMZ) were cultured in 12-well tissue culture plates (Corning) at a density of 0.5 × 10^6^ to 1.0 × 10^6^ cells/ml in minimal essential medium alpha (MEM-alpha) (Gibco) with 20% fetal bovine serum (FBS; HyClone, GE Healthcare), 1% GlutaMAX (Gibco), 10% conditioned supernatant from renal carcinoma cell line 5637 (ACC-35; DSMZ), 100 U/ml penicillin, and 100 μg/ml streptomycin (Gibco) at 37°C with 5% CO_2_. We obtained MUTZ-3-derived Langerhans cells (muLCs) by differentiation of MUTZ-3 cells for 10 days in 100 ng/ml granulocyte-macrophage colony-stimulating factor (GM-CSF; GenWay Biotech), 10 ng/ml transforming growth factor β (TGF-β; R&D Systems), and 2.5 ng/ml TNF-α (R&D Systems) as described previously (22, 23). The phenotype of differentiated muLCs was verified by surface staining of CD34 (clone 581; BD Biosciences), CD1a (clone HI149; BD Biosciences), and CD207 (clone DCGM4, Beckman Coulter) using the respective antibodies and analysis by flow cytometry.

THP1 cells (TIB-202; ATCC) transduced with a lentiviral langerin construct or empty vector (EV) were cultured in RPMI (Lonza) supplemented with 5% FBS (Biowest), 1% GlutaMAX, 100 U/ml penicillin, and 100 μg/ml streptomycin (Gibco) at 37°C with 5% CO_2_.

### Isolation of primary human Langerhans cells.

LCs were isolated from human skin as described previously (38). In short, skin grafts were obtained using a Zimmer Dermatome and incubated in medium supplemented with dispase II (1 U/ml; Roche Diagnostics), after which epidermal sheets were separated from the dermis and cultured for 3 days. After incubation, migrated LCs were harvested and further purified using a Ficoll gradient (Axis-shield). Isolated LCs were routinely 90% pure (CD1a^+^ Langerin^+^) and were frozen in Iscove’s modified Dulbecco’s medium (IMDM; Thermo Fisher) supplemented with 20% FBS and 10% dimethyl sulfoxide (DMSO). Before use, LCs were thawed by dropwise addition of cold IMDM with 10% FBS, washed twice, and incubated in IMDM with FBS for 2 h at 37°C with 5% CO_2_ to recover.

### Creation of GFP-expressing S. aureus.

To create GFP-expressing bacteria, the S. aureus Newman wild-type and Newman Δ*spa* Δ*sbi* strains were transformed as described previously with pCM29, which encodes superfolded green fluorescent protein (sGFP) driven by the *sarA*P1 promoter (56, 57). In short, competent S. aureus cells were electroporated with pCM29 isolated from Escherichia coli DC10B with a Bio-Rad Gene Pulser II (100 ohm, 25 μF, 2.5 kV). After recovery, bacteria were selected on Todd-Hewitt agar supplemented with 10 μg/ml chloramphenicol. A single colony was grown in THB with 10 μg/ml chloramphenicol under the usual growth conditions. Bacterial expression of GFP was verified by confocal laser scanning microscopy (SP5; Leica).

### Gamma irradiation of S. aureus.

Gamma-irradiated stocks of S. aureus strains were made by harvesting cultures in mid-exponential growth phase by centrifugation (4,000 rpm, 8 min) and concentrated 10× in phosphate-buffered saline (PBS; Lonza) with 17% glycerol (VWR), frozen at −70°C, and exposed to 10 kGy of gamma radiation (Synergy Health, Ede, The Netherlands). Loss of viability of irradiated S. aureus was verified by plating. A nonirradiated aliquot that underwent the same freezing procedure was used to determine the number of CFU of the irradiated stocks.

### Lentiviral transduction.

A TrueORF sequence-validated cDNA clone of human CD207 (OriGene Technologies) was amplified by PCR using Phusion polymerase (Thermo Fisher) and primers hLangerin-Fw (5′-GAGCTAGCAGTATTAATTAACCACCATGACTGTGGAGAAGGAG-3′) and hLangerin-FLAG-Rv (5′-GTTTCTTTTCATTTGTAAGCGACCCTATGTCCCATCAGAACCGGACTACAAAGACGATGACGACAAGTGAGCATGCATCCTAACCGGTAC-3′) (IDT). The PCR amplicon was cloned in a BIC-PGK-Zeo-T2a-mAmetrine;EF1A construct by Gibson assembly (NEB) according to the manufacturer’s instructions. The langerin-encoding vector and an empty vector (EV) control were introduced into THP1 cells by lentiviral transduction, as described by van de Weijer et al. (58). In short, lentivirus was produced by HEK293T cells (CRL-3216; ATCC) in 24-well plates using standard lentiviral production protocols and third-generation packaging vectors. After 3 to 4 days, the supernatant containing the viral particles was harvested and stored at −70°C to kill any remaining cells. Approximately 50,000 THP1 cells were transduced by spin infection (1,000 × *g*, 2 h, 33°C) using 100 μl supernatant supplemented with 8 μg/ml Polybrene (Santa Cruz Biotechnology). Complete medium was added after centrifugation, and cells were selected 3 days postinfection by 100 μg/ml Zeocin (Gibco). Cellular expression of langerin was verified by antibody staining of langerin (clone DCGM4; Beckman Coulter) and measured using flow cytometry.

### Bacterial binding assays.

To test binding of bacteria to cells, 10^5^ human primary LCs or THP1-EV or THP1-langerin cells were incubated with GFP-expressing S. aureus Newman or GFP-expressing S. aureus Newman Δ*spa* Δ*sbi* cells at bacterium-to-cell ratios from 1 to 8 in TSM buffer (2.4 g/liter Tris [Roche], 8.77 g/liter NaCl [Sigma-Aldrich], 294 mg/liter CaCl_2_⋅2H_2_O [Merck], 294 mg/liter MgCl_2_⋅6H_2_O [Merck] at pH 7.4) with 0.1% bovine serum albumin (BSA; Merck) for 30 min at 4°C. Binding was blocked by 15 min of preincubation with 10 μg/ml mannan (Sigma-Aldrich), 50 mM GlcNAc (Serva), or 20 μg/ml anti-langerin blocking antibody (clone 10E2; Sony Biotechnology). Cells were washed once with TSM–1% BSA, fixed in 1% formaldehyde (Brunschwig Chemie) in PBS, and measured by flow cytometry. Primary LC-S. aureus cell binding data were normalized to the maximum binding level per donor.

### Production of recombinant langerin extracellular domains.

The extracellular domains (ECDs) of truncated human langerin (residues 148 to 328) and mouse langerin (residues 150 to 331) were recombinantly expressed from codon-optimized constructs containing a C-terminal tobacco etch virus (TEV) cleavage site followed by Strep-tag II cloned into pUC19 and pET30a (EMD Millipore) expression vectors as described previously (28). Recombinant human and murine ECDs were insolubly expressed in Escherichia coli BL21(DE3), solubilized in 6 M guanidinium hydrochloride in 100 mM Tris (pH 8) with 1 mM dithiothreitol (DTT), refolded by dialysis against Tris-buffered saline (pH 7.5) containing 10 mM CaCl_2_, and purified via mannan-coupled Sepharose beads (Sigma-Aldrich). Bound protein was eluted with Tris-buffered saline (pH 7.5) containing 5 mM EDTA. Protein concentrations were determined through absorbance at 280 nm using the calculated molar extinction coefficients of 56,170 M^−1 ^cm^−1^ for the human langerin ECD and 56,170 M^−1 ^cm^−1^ for the murine ECD. The proteins were fluorescently labeled by slowly adding 100 μl of 1 mg/ml fluorescein isothiocyanate (FITC; Thermo Fisher) in DMSO to 2 ml of a 2-mg/ml protein solution in HEPES-buffered saline (pH 7.2) containing 20 mM d-mannose (Sigma-Aldrich) and 5 mM CaCl_2_. After being stirred for 90 min at room temperature, the reaction was quenched by addition of 50 mM ethanolamine (pH 8.5; Sigma-Aldrich). Unreacted dye molecules were removed by buffer exchange using a Zeba spin column (Thermo Fisher), and active protein was purified over a mannan affinity column as described above. All chemicals used for the production of recombinant langerin extracellular domains were obtained from Carl Roth unless indicated otherwise.

### Langerin binding assay.

Bacteria in the mid-exponential growth phase were harvested by centrifugation (4,000 rpm, 8 min) and resuspended at an OD_600_ of 0.4 in TSM buffer with 0.1% BSA. Bacteria were incubated with 1 to 50 μg/ml recombinant langerin-FITC (human or mouse) for 30 min at 37°C with agitation, washed once with TSM–1% BSA, fixed in 1% formaldehyde, and analyzed by flow cytometry.

### muLC stimulation.

We stimulated 5 × 10^4^ muLCs with gamma-irradiated S. aureus USA300 WT, USA300 Δ*tarM*, or USA300 Δ*tarS* at bacterium-to-cell ratios of 0, 1, 10, and 50 in IMDM with 10% FBS. After 24 h, supernatants were collected by centrifugation (300 × *g*, 10 min, 4°C) and stored at −150°C until further analysis, and cells were washed once in PBS–0.1% BSA. Expression levels of the activation and maturation markers were determined by flow cytometry using the antibodies CD80 (clone 2D10), CD83 (clone HB15e), and CD86 (clone IT2.2) (all from Sony Biotechnology) and their corresponding isotype controls (BD Biosciences).

### Cytokine assays.

The initial IL-8 and IL-12p70 concentrations (Fig. 1C), as well as IFN-γ concentrations (relating to Fig. 3B), were determined by enzyme-linked immunosorbent assay (ELISA; Sanquin for IL-8 and Thermo Fisher for IL-12p70 and IFN-γ) according to the manufacturers’ instructions. Concentrations of IL-4, IL-6, IL-8, IL-10, IL-12p70, IL-23p19, and TNF-α cytokines (Fig. 3B) were determined by Luminex xMAP assay (Luminex Corporation), performed by the Multiplex Core Facility, UMC Utrecht, Utrecht, The Netherlands.

### Flow cytometry.

Flow cytometry was performed on a FACSVerse (BD Biosciences). Per sample, 10,000 events within the set gate were collected. Data were analyzed using FlowJo 10 (FlowJo, LLC).

### Epicutaneous murine infection model.

All mice were housed in a specific-pathogen-free facility under standard conditions at the University of Pittsburgh, Pennsylvania, USA. As described previously, 6- to 10-week-old sex-matched wild-type C57BL/6 mice (obtained from Jackson Laboratories) and huLangerin-DTR mice (29) were first anesthetized with a mixture of ketamine and xylazine (100/10 mg/kg body weight), shaved on the back with electric clippers, and chemically depilated with Nair hair removal cream (Church & Dwight) according to the manufacturer’s instructions, and the stratum corneum was removed by 15 strokes of 220-grit sandpaper (3M) (9, 30). Previous data show that approximately 50% of the stratum corneum is removed by this procedure, while the epidermal layer is left intact, based on hematoxylin staining of sections (9). After 24 h, the mice were epicutaneously inoculated with 50 μl sterile PBS with or without 1.0 × 10^7^ CFU of S. aureus USA300 Δ*tarM* or S. aureus USA300 Δ*tarS*, which were grown overnight at 37°C in THB. Forty hours postinfection, the mice were sacrificed and skin sections of 1 cm^2^ were collected. The sections were either (i) homogenized, serially diluted in sterile PBS, grown overnight on THB-agar plates or MRSA (methicillin-resistant S. aureus)-specific CHROMagar MRSA-II plates (BD) at 37°C to quantify CFU, (ii) homogenized and processed for RNA extraction, or (iii) fixed in 1% formalin in PBS. The fixed tissue sections were embedded in paraffin, cut, stained with hematoxylin and eosin, and digitalized (Hamamatsu NanoZoomer) by the Department of Pathology, UMC Utrecht, Utrecht, The Netherlands, and subsequently analyzed using NDP.view2.6.13 (Hamamatsu).

### Gene expression analysis.

Whole skin was homogenized and processed for extraction and isolation of RNA, using TRIzol reagents (Thermo Fisher), following the manufacturer’s instructions. RNA was quantified using a standard Nanodrop, and cDNA was obtained using high-capacity cDNA reverse transcriptase (Thermo Fisher). Quantitative PCR on cDNA was performed using TaqMan gene expression master mix and TaqMan gene expression assays for *Il17*, *Il6*, *Cxcl1*, *Cxcl2*, *Il10*, *Ifng*, and *Gapdh* (Thermo Fisher) on a StepOnePlus real-time PCR system (Applied Biosystems), according to the manufacturers’ instructions. Log_2_-transformed fold change of transcripts was calculated from threshold cycle (ΔΔ*C_T_*) values relative to *Gapdh* expression, normalized for the PBS mock control.

### Statistical analysis.

Data are presented as the geometric mean or percentage of positive cells (flow cytometry), mean concentration ± standard error of the mean (SEM) (cytokine assays), or log_2_ fold change ± standard deviation (SD) (real-time PCR). Statistical analyses were performed using Graphpad Prism 7.02 (GraphPad Software). Data were analyzed by unpaired two-tailed *t* test or one-way analysis of variance (ANOVA) followed by Dunnett’s multiple-comparison test, except as follows. Primary LCs and THP1-langerin dose-response curves, were tested using a two-way ANOVA followed by Dunnett’s multiple-comparison test, Langerin-FITC concentration curves were tested against wild-type langerin-FITC using a two-way ANOVA followed by Tukey’s multiple-comparison test, and gene expression data were tested using one-way ANOVA followed by Sidak’s multiple-comparison test. *P* < 0.05 was considered significant, and *P* values are indicated in the respective figures.

### Data availability.

The data that support these findings are available from the corresponding author upon request.
