# Supplementary figures and images for: Langerhans Cells Sense Staphylococcus aureus Wall Teichoic Acid through Langerin To Induce Inflammatory Responses
Source: mBio. 2019 May 14;10(3):e00330-19. doi: 10.1128/mBio.00330-19 (PMC6520447; doi:10.1128/mBio.00330-19)

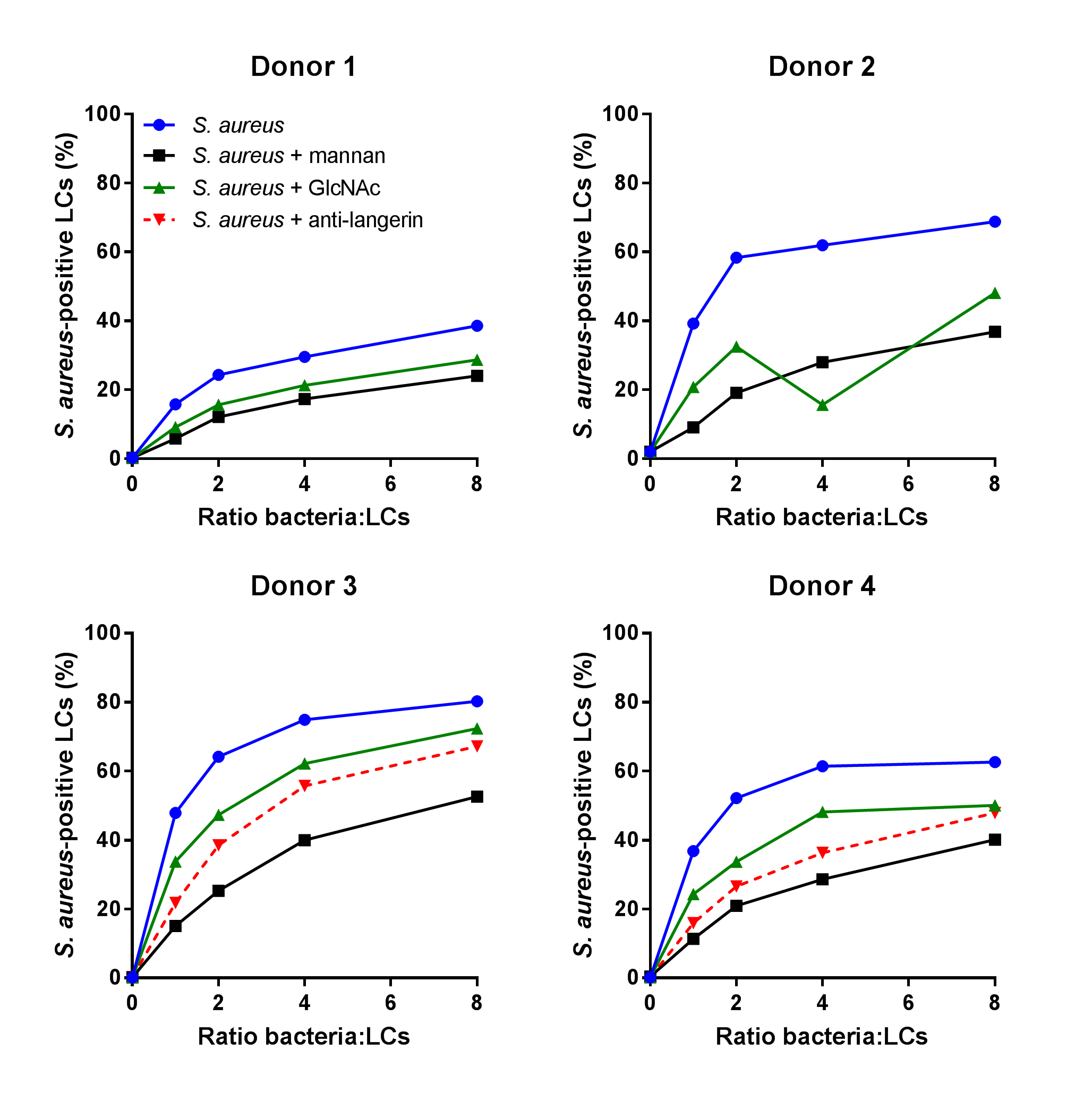

Supplement: FIG S1 [file mBio.00330-19-sf001.tif]

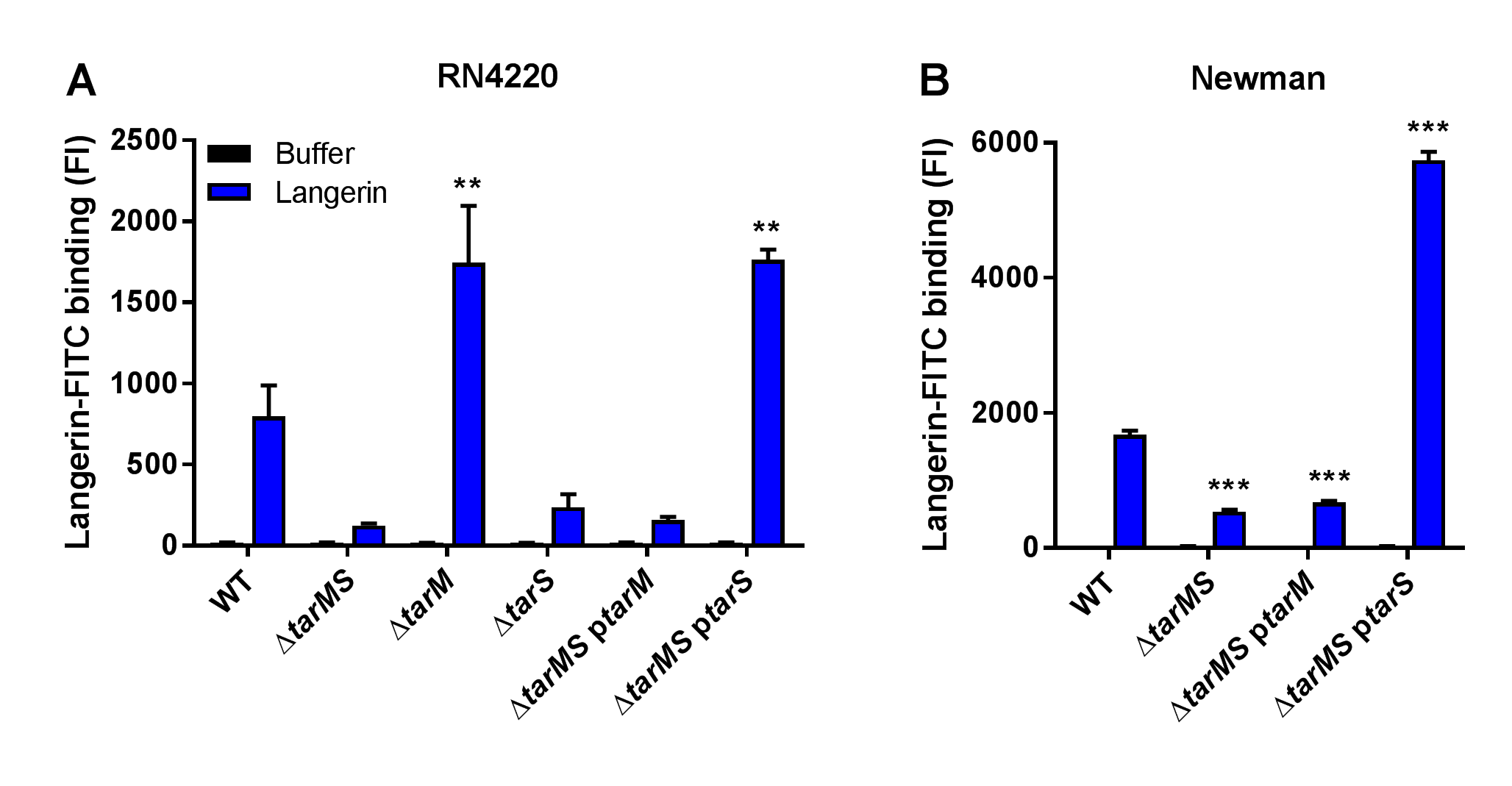

Supplement: FIG S2 [file mBio.00330-19-sf002.tif]

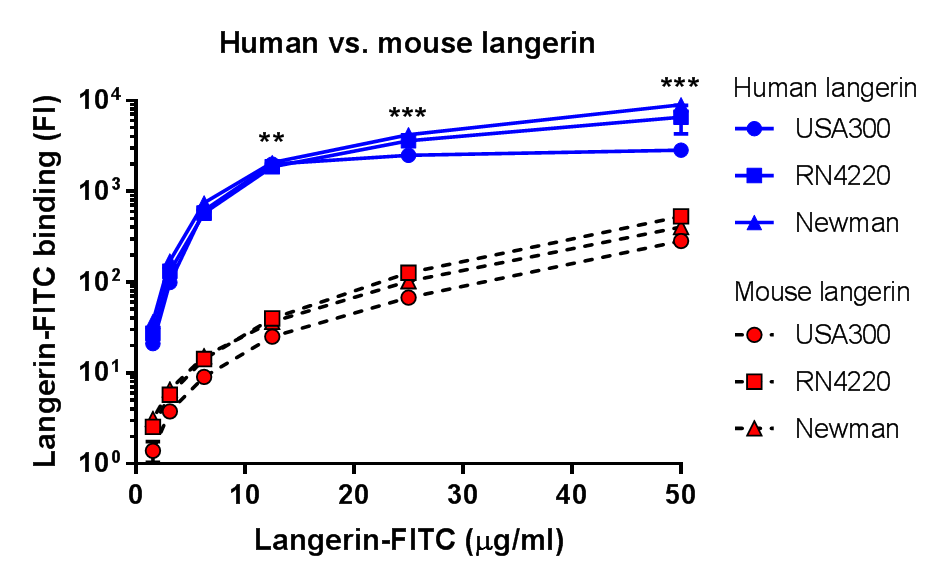

Supplement: FIG S3 [file mBio.00330-19-sf003.tif]

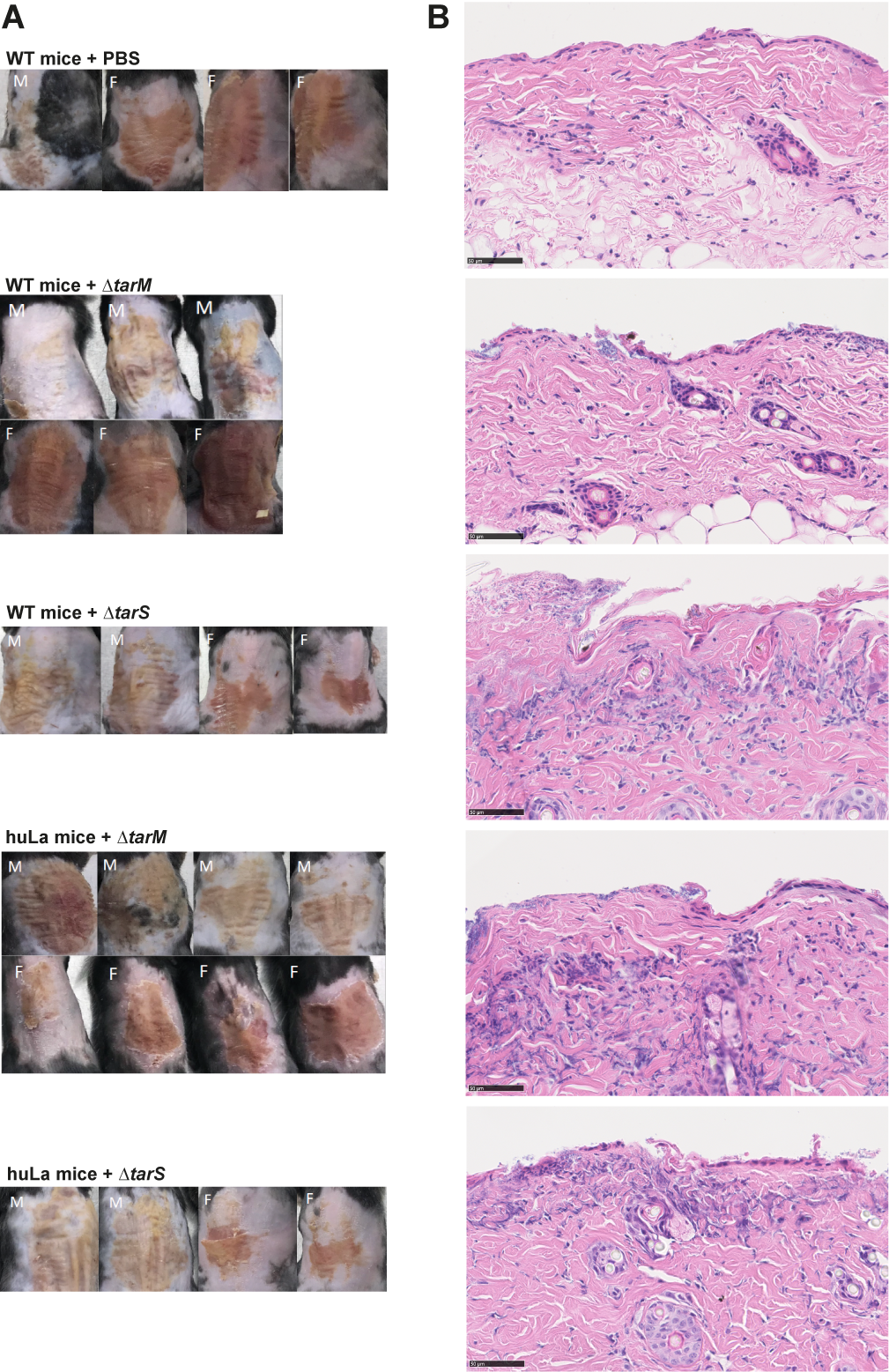

Supplement: FIG S4 [file mBio.00330-19-sf004.tif]
