# Supplementary material for: Langerhans Cells Sense Staphylococcus aureus Wall Teichoic Acid through Langerin To Induce Inflammatory Responses
Source: mBio. 2019 May 14;10(3):e00330-19. doi: 10.1128/mBio.00330-19 (PMC6520447; doi:10.1128/mBio.00330-19)
Supplement: TABLE S1 [file mBio.00330-19-st001.docx]

## Supplementary Table S1. Bacterial strains used in this study.

| **Strain** | **Source** |
| --- | --- |
| *S. aureus* Newman wild type (ST254, CC8) | ATCC, Cat#13420 |
| *S. aureus* Newman Δ*tarM*Δ*tarS* | Winstel et al., 2013 (15) |
| *S. aureus* Newman Δ*tarM*Δ*tarS* pRB474-*tarM* | Winstel et al., 2013 (15) |
| *S. aureus* Newman Δ*tarM*Δ*tarS* pRB474-*tarS* | Winstel et al., 2013 (15) |
| *S. aureus* Newman Δ*spa*Δ*sbi* | Sibbald et al., 2010 (49) |
| *S. aureus* Newman pCM29 (sGFP) | This paper |
| *S. aureus* Newman Δ*spa*Δ*sbi* pCM29 (sGFP) | This paper |
| *S. aureus* USA300 wild type (NRS384, ST8, CC8) | NARSA strain collection |
| *S. aureus* USA300 Δ*tarM* | Winstel et al., 2015 (11) |
| *S. aureus* USA300 Δ*tarS* | Brown et al., 2012 (13) |
| *S. aureus* USA300 Δ*tarM*Δ*tarS* | Winstel et al., 2015 (11) |
| *S. aureus* USA300 Δ*tarM*Δ*tarS* pRB474-*tarM* | Winstel et al., 2015 (11) |
| *S. aureus* USA300 Δ*tarM*Δ*tarS* pRB474-*tarS* | Winstel et al., 2015 (11) |
| *S. aureus* RN4220 wild type (ST8, CC8) | Kreiswirth et al., 1983 (50) |
| *S. aureus* RN4220 Δ*tarM* | Winstel et al., 2013 (15) |
| *S. aureus* RN4220 Δ*tarS* | Winstel et al., 2013 (15) |
| *S. aureus* RN4220 Δ*tarM*Δ*tarS* | Winstel et al., 2013 (15) |
| *S. aureus* RN4220 Δ*tarM*Δ*tarS* pRB474-*tarM* | Winstel et al., 2013 (15) |
| *S. aureus* RN4220 Δ*tarM*Δ*tarS* pRB474-*tarS* | Winstel et al., 2013 (15) |
| *S. aureus* 82086 wild type (ST398, CC398) | Winstel et al., 2015 (15) |
| *S. aureus* 82086 Δ*tarS* | Li et al., 2015 (17) |
| *S. aureus* PS66 wild type (NCTC 8288, ST39, CC30) | Udo Bläsi, Vienna |
| *S. aureus* PS66 Δ*tarS* | Li et al., 2015 (17) |
| *S. aureus* MW2 wild type (ST1, CC1) | CDC, 1999 (51) |
| *S. aureus* MRSA252 wild type (ST36, CC30) | Tim Foster, Dublin |
| *S. aureus* MSSA467 wild type (ST1, CC1) | Tim Foster, Dublin |
| *S. aureus* Wood46 wild type (CC97) | ATCC, Cat#10832 |
| *S. aureus* Mu50 wild type (ST5, CC5) | CDC, 1999 (51) |
| *S. aureus* P68 wild type (ST25, CC25) | Udo Bläsi, Vienna |
| *S. aureus* NRS22 wild type (USA600, ST45, CC45) | NARSA strain collection |
| *S. aureus* NRS184 wild type (ST22, CC22) | NARSA strain collection |
| *S. aureus* JH1 wild type (ST105, CC5) | Mwangi et al., 2007 (52) |
| *S. aureus* N315 wild type (ST5, CC5) | NARSA strain collection |
| *S. aureus* PS187 wild type (ST395, CC395) | ATCC, Cat#15564 |
| *S. aureus* ED133 wild type (ST133, CC133) | Guinane et al., 2010 (53) |
| *S. aureus* Lowenstein wild type | ATCC, Cat#49521 |
| *S. capitis* ATCC27840 wild type | ATCC, Cat#27840 |
| *S. carnosus* TM300 wild type | Rosenstein et al., 2009 (54) |
| *S. epidermidis* 1457 wild type | Mack et al., 1992 (55) |
| *S. lugdunensis* SL13 wild type | Chassain et al., 2012 (56) |
| *S. pseudintermedius* ED99 wild type | Ben Zakour et al., 2011 (57) |
| *S. saprophyticus* ATCC 35552 wild type | ATCC, Cat#35552 |
| *S. simulans* ATCC 27848 wild type | ATCC, Cat#27848 |
| *E. coli* DC10B | Monk et al., 2012 (58) |
| *E. coli* BL21(DE3) | Thermo Fisher, Cat#C6000-03 |
